# Motor strength as a feature of concepts and visual representations

**DOI:** 10.3389/fpsyg.2024.1164836

**Published:** 2024-02-12

**Authors:** Omid Khatin-Zadeh, Jiehui Hu, Danyal Farsani

**Affiliations:** ^1^School of Foreign Languages, University of Electronic Science and Technology of China, Chengdu, China; ^2^Department of Teacher Education, Norwegian University of Science and Technology, Trondheim, Norway

**Keywords:** gesture, motion, motor strength, motor system, visual representation

## Abstract

In this article, we define *motor strength* as the extent to which a concept is associated with body movements and the motor system that guides body movements. We extend this notion to one of the features of visual representations of some concepts and discuss the role of the motor system in understanding concepts and visual representations that have a significant degree of motor strength. It is suggested that when a concept is understood in its *literal* sense, the employment of the motor system and gestures in processing that concept depends on its degree of motor strength. If a concept is understood in its *metaphorical* sense, the employment of the motor system and gestures is dependent on the degree of motor strength of the base of the metaphor through which that concept is understood. The degree of motor strength of a concept relies on its motor affordances and its associations with people’s past experiences. Because the motor system plays an essential role in the grounding of many abstract concepts in the physical environment, the notion of motor strength can help psychologists acquire a clearer understanding of how concepts with varying degrees of motor strength are grounded in the physical environment.

## 1 Introduction

In the past two decades, embodied theories of cognition have been the subject of a large body of research and discussions among researchers working in various fields (e.g., Gianelli et al., 2013; Robinson and Thomas, 2021; Dove et al., 2022; Khatin-Zadeh et al., 2022a; Khatin-Zadeh, 2023). All versions of embodied cognition share the assumption that people’s sensorimotor systems play a crucial role in their understanding of concepts (e.g., Glenberg et al., 2008; Lakoff, 2008, 2014; Binder and Desai, 2011; Kiefer and Pulvermüller, 2012; Hauk and Tschentscher, 2013; Lambon-Ralph, 2013; Zwaan, 2014; for a review, see Khatin-Zadeh et al., 2021). Embodied theories of cognition hold that concrete and even abstract concepts are grounded through our sensorimotor systems (e.g., Khatin-Zadeh and Yazdani-Fazlabadi, 2023). People perceive concepts’ characteristics through the sensorimotor systems, which enables them to create sensorimotor representations of the concepts in the neural networks.

Any concept may have varying degrees of sensory features (Villani et al., 2019). Some concepts are highly visual, whereas others are strongly auditory, haptic, gustatory, or olfactory. Some concepts may be strong in more than one sensory modality. For example, the concept of *paper* is highly visual and haptic, as it can be easily seen and touched. But, it does not have a significant degree of auditory, gustatory, and olfactory associations. In contrast, the concept of *scream* is highly auditory, as it can be easily perceived through our ears and auditory system. But, it does not have a high degree of visual, haptic, gustatory, and olfactory associations. A question raised here is “how can degrees of sensory strength of a concept in various modalities be determined?” If there is a criterion for determining degrees of sensory strength of a concept in various modalities, this will allow us to determine which sensory modalities are more significant in the embodied processing of that concept and its grounding through sensory systems.

Several studies have used objective methods for measuring perceptual strength of concepts in some languages (e.g., Filipović Ðurğević et al., 2016; Miklashevsky, 2018; Chen et al., 2019). Speed and Majid (2017) measured perceptual strength (visual, auditory, haptic, gustatory, and olfactory associations) of 485 Dutch nouns. They found that the nouns were highly multimodal, being most dominant in vision and least dominant in olfaction. In addition, some nouns were perceptually strong in both olfaction and gustation, whereas some other nouns were perceptually strong in both vision and touch. A more comprehensive study measured degrees of sensorimotor strength of 4,000 English words (Lynott et al., 2020). In that study, sensory and action strengths of concepts across six perceptual modalities (touch, hearing, smell, taste, vision, and interoception) and five action effectors (mouth, hand, foot, head, and torso) were measured objectively. Participants of the study were given lists of words and were asked to rate how much they experienced concepts using six perceptual senses or five action effectors. The rating scales ranged from 0 (not experienced at all with that sense or action effector) to 5 (experienced greatly with that sense or action effector). For each word, the mean of perceptual strength in each sense and the mean of action effector strength in each effector were calculated, resulting in what is known as the Lancaster Sensorimotor Norms (LSN). The data provided in the LSN database offer some criteria for determining the degrees of sensory and action effector strengths of concepts.

In the following section, sensory and action effector strengths of several concepts based on data provided in the LSN (Lynott et al., 2020) are discussed to prepare the ground for presenting the main point of our paper. That point is that motor strength plays a role in perception of both concrete and abstract concepts and representations.

## 2 Sensory and action effector strengths of concepts

According to the data that have been provided in the LSN (Lynott et al., 2020), different concepts have a variety of sensory and action effector strengths. Degrees of sensory and action effector strength of a concept are dependent on the physical characteristics of that concept and its association with actions of various parts of the body. For example, the concept of *pen* has strong visual and haptic strengths (Kim et al., 2020). In LSN, degrees of visual and haptic strength of this concept are 4.211 and 4.316, respectively. Because physical characteristics of a pen can be perceived primarily through the visual and haptic senses, it is strong in those senses. This concept is much weaker in other sensory modalities, as physical characteristics of pen have little effect on the auditory, gustatory, and olfactory senses. In the LSN, the degrees of auditory, gustatory, and olfactory strength of the concept *pen* are 0.526, 0.053, and 0.158.

Because people often use their hands to work with a pen, it has strong associations with *hand*. According to the data presented in LSN, degree of hand action strength of this concepts is 4.238, which is very high. Degrees of head, foot, and mouth action strength of this concept are 2.286, 0.238, and 0.333, respectively. The latter two values are particularly low because the feet and mouth are rarely used to write with a pen. The data show that *pen* has a significant degree of head action strength, although much less than hand action strength. People primarily use their hands to work with a pen. However, the movements of hands are often accompanied by some degree of head movements when writing; people may continuously change their head position to see what they are writing or to take some rest. In fact, there is some coordination between the movements of hand and head when a pen is used to write something. This coordination can explain why the concept of *pen* has a significant degree of head action strength. In other words, degree of action strength of a concept in a certain part of the body depends on the degree of association of that body part with the concept. When several parts of the body are used simultaneously to work with an object, that object has significant associations with all those body parts, but one specific part may take the dominant role and have the strongest association with that object. In the case of *pen*, the dominant role is played by the hands but the head also plays a significant role.

Another example can further clarify the point. The concept of *walk* has a strong visual strength, because the action of walking can be easily perceived through vision. According to the data in the LSN, visual strength of this concept is 3.118. *Walk* is much weaker in auditory, gustatory, and olfactory strengths (0.765, 0, and 0.176, respectively). Degree of haptic strength of *walk* is 1.588, which is higher than the auditory, gustatory, and olfactory strengths of this concept but lower than its visual strength. The action of walking can be felt when a person’s feet touch the ground. Therefore, to some extent, the action of walking can be perceived through the sense of touch. This explains why the concept of *walk* has a significant degree of haptic strength, compared to its auditory, gustatory, and olfactory strengths. Because feet play the most important role in the action of walking, foot action strength of this concept is very high (4.947). Mouth action strength of this concept is very low (0.526), as the mouth does not play a significant role in the action of walking. Degrees of hand and head action strength of this concept are higher than its mouth action strength but much lower than its foot action strength. The action of walking involves some degree of hand and head movements, which explains why the concept of *walk* has some degree of hand and head action strength.

According to embodied theories of cognition, when someone talks about, thinks about, or cognitively processes a concept, their past sensorimotor experiences in dealing with that concept or the object(s) that represents that concept are activated (e.g., Rizzolatti and Craighero, 2004; Gallese and Lakoff, 2005; Iacoboni et al., 2005; Boulenger et al., 2009; Rizzolati and Sinigaglia, 2010; Harpaintner et al., 2020). Therefore, when the concept of *pen* is processed, the sensorimotor experiences in dealing with pens, which are mostly visual and haptic, are activated in the mind. This activation primarily takes place in the neural networks of the visual and haptic systems. Specifically, if a concept has a high action effector strength in a certain part of the body, that part of the body and that part of the motor system that controls it play key roles in grounding and processing that concept. Therefore, according to embodied theories of cognition, when the concept is talked about, thought about, or processed, the area of the motor system that controls that part of the body is activated.

Based on what was mentioned above, an extended definition of *motor strength* of a concept is introduced here (Khatin-Zadeh et al., 2022b,2023b). Specifically, motor strength of a concept is the extent to which the concept is associated with motion and body movement. If a concept is essentially a motion event or has direct association with motion events or body movements, it has a high motor strength. Even concepts that are not themselves motions or are not directly associated with motion events may have some degree of motor associations. In other words, even an indirect association or metaphorical association between a concept and motion events (or body movements) gives that concept some degree of motor strength.

The values of action effector strength presented in the LSN provide one criterion for determining motor strengths of concepts. However, there may be other criteria for this. The number of gestures that people use when talking about a concept can be another criterion for determining degree of motor strength of that concept. In daily experiences, it can be observed that some concepts have a strong tendency to be accompanied with gestures when being talked about. For example, people tend to use gestures and body movements when they talk about the concept of *sprint*. This concept includes a set of body movements. Even a concept such as *anger*, which is not a motion event or body movement, is used with a significant number of gestures. In this case, the association between the concept of *anger* and body movement is metonymic and metaphorical (Kövecses, 2005, 2013). It is metonymic because the emotional state of anger is often accompanied with rapid body movement in many people. It is metaphorical because the emotional state of anger is metaphorically described as an upward movement in many cases. These metonymic and metaphoric associations between the concept of anger and motions or body movements (Kövecses, 2005, 2013) give this concept some degree of motor strength.

In teaching and learning sciences, teachers and students widely use gestures. This means that not only daily concepts but also scientific concepts can have motor strength, and this motor strength is realized in gestures that are used to talk about them. Results of many studies have demonstrated that gestures enhance the process of teaching and learning sciences (e.g., Alibali and Nathan, 2012; Alibali et al., 2014; Martinez-Lincoln et al., 2018; Rosa and Farsani, 2021; Farsani and Villa-Ochoa, 2022; Khatin-Zadeh et al., 2023a). Many of these concepts such as those discussed in mathematics (numbers, limit of function, continuity, etc.) are highly abstract. However, results of past studies have shown that gestures are powerful tools for understanding even these highly abstract concepts (e.g., Núñez, 2005; Alibali and Nathan, 2012; Marghetis and Núñez, 2013). Therefore, it can be suggested that abstract concepts can have some degree of motor strength, and this motor strength is realized in gestures that are used to talk about these concepts. Even it can be assumed that abstract concepts that are often accompanied by a higher number of gestures have higher degrees of motor strength. Abstract concepts can become strongly motoric through metaphors. We will discuss this process in the following section.

## 3 Various types of motor strength

Some concepts are inherently motions, body movements, or are directly associated with motions or body movements. The concepts *of run, walk, sprint, jump, grasp*, and *fly* are examples of these concepts. Such concepts have strong degrees of motor strength. Some concepts are inherently static but have indirect associations with motion events or body movements. The concepts of *pencil, shoe, road*, and *screwdriver* are examples of such concepts. A pencil is a static object, but body movements occur when writing with it. A shoe is also a static object, but shoes are used to walk and run. The concept of road is also static, but it has a certain degree of motor strength or motor association because people have had the experience of walking on a road or can imagine moving on a road, as a road affords movement. These associations give *road* some degree of motor strength, as processing the concept of *road* can lead to the simulation of motor experiences that are associated with roads.

Some concepts are inherently static but are metaphorically described in terms of motion events. This metaphorical description gives such concepts some degree of motor strength. For example, the concept of *time* is abstract and non-motion. But, in many metaphorical sentences, it is described as a moving object. This metaphorical description gives the concept of *time* some degree of motor strength. When people metaphorically talk about the fast movement of time, they may use a gesture to show this fast movement. Using a gesture to show the fast metaphorical movement of time shows that this abstract concept has some degree of motor strength. To take another example, the concept of *love* is inherently abstract and does not have any semantic dimension related to body movement. However, in some metaphorical statements, this concept is metaphorically understood in terms of movement. The metaphors *we have come a long way on the road of happiness*, and *the lovers were flying in the clouds* are two examples that describe love relationships in terms of movement. Although the concept of *love* does not inherently have any direct semantic association with body movement, such metaphors describe it in terms of body movement to ground it through the sensorimotor system.

In the metaphorical description of concepts, one specific but important group of cases is the description of concepts in terms of visual representations. In many scientific and even daily discussions, graphs, diagrams, tables and other visual tools are used to represent concepts and relations among concepts (e.g., Radford, 2009; Font et al., 2010; Glazer, 2011). Because many concepts are abstract, they are often described in terms of visually perceivable tools to provide a better understanding of them, which is a widely used strategy in many scientific disciplines (Duijzer et al., 2019). Visual tools help people to ground abstract concepts in easily perceivable representations. In this way, abstract concepts are grounded in concrete environment and are embodied through their concrete representations. The ways through which visual representations of concepts can help people to acquire a better understanding of concepts and ideas have been discussed in many works (for a review, see Duijzer et al., 2019). Here, we specifically focus on the employment of the motor system (as a cognitive resource) through visual representations of abstract concepts. Here, we focus on this specific group of metaphorical descriptions to offer a picture of how the motor system can help to improve a person’s understanding when an abstract concept is metaphorically described in terms of a strongly motorized visual representation.

Our main assertion is that when a concept is metaphorically represented in terms of a motorized visual representation, the motor system, as one of the components of human cognitive resources, is actively employed to process that concept. To support this assertion, evidence provided by a number of studies that have suggested that how visual representations of concepts can have some degree of motor strength is reviewed. In the next section, metaphorical descriptions of abstract concepts in terms of visual representations are discussed. This will be followed by discussing activation of the motor system as a result of looking at static images and the role of motor strength in understanding a concept in terms of a visual representation.

## 4 Understanding a concept in terms of a visual representation of that concept

When an abstract concept is metaphorically described in terms of a visual representation, new channels of grounding are opened, and new resources are employed to understand that concept. In this way, an amodal representation of the concept is understood in terms of or through a modal or multimodal representation of the concept (e.g., Marmolejo-Ramos et al., 2017; Tirado et al., 2018; Khatin-Zadeh et al., 2021). In fact, the reason why people transform an abstract representation of a concept into a concrete representation is to acquire a better understanding of the original abstract representation through the mediatory concrete representation (Khatin-Zadeh et al., 2022b). This is what happens in the metaphorical description of an unfamiliar abstract concept in terms of a familiar concrete concept (Lakoff and Johnson, 2003). In the process of transforming the abstract representation into a concrete representation, the two representations must be isomorphic. That is, the information that is true about the concrete representation must also be true about the abstract representation. One specific property of the concrete representation that can play a key role in the process of grounding is its degree of motor strength. If the concrete representation of the concept has a high degree of motor strength, the motor system plays an essential role in the grounding of the abstract representation through the concrete representation. In fact, the higher the degree of motor strength of the concrete representation, the higher the degree of motor-system involvement in the process of grounding and understanding the abstract representation.

In the metaphorical processing of abstract concepts in terms of concepts that have some significant degree of motor strength, the motor system can be employed as an extra cognitive resource. In this way, even highly abstract concepts can be understood through the sensorimotor system (Borghi et al., 2017; Borghi, 2020). In many scientific discussions, some concepts and relations among concepts are described in terms of visual representations such as graphs, diagrams, tables, coordinate systems, and vectors. These visual representations help people to metaphorically understand highly abstract scientific concepts. These metaphors can be called scientific metaphors, which are not inherently different from linguistic metaphors because they are used to describe a target concept in terms of a base concept. Visual representations of scientific concepts significantly facilitate the process of understanding such concepts. Visual representations of concepts have a high degree of concreteness. Therefore, they can be used as mediators to ground abstract concepts through the sensorimotor system. An example of visual representation of a scientific concept is a curve that represents the visual representation of a mathematical function in the Cartesian coordinate system. A curve as the graphical representation of a mathematical function may have some degree of motor strength, as a curve can be conceived as the frozen version of a hand movement that has created it. One can imagine the process of creating the curve with a hand movement and even imagine a gesture that depicts that curve in the space.

The process of understanding a given concept with some degree of motor strength can involve the activation of the motor system. The extent of motor system involvement is affected by many factors, including conditions of the task, the intention of the individual, and the context in which the concept is processed. Although handwritten letters and paintings are static, they can have some degree of motor strength and activate the motor system (e.g., Longcamp et al., 2011; Umiltà et al., 2012). The motor strength of these works may be associated with the hand movements that have created them. Therefore, motor strength of a concept is dependent on people’s knowledge of that concept and their past experiences with it. Handwritten letters, paintings, mathematical graphs, and many other things are essentially static, but their formations involve hand movement. The history of formation can be one semantic dimension of any concept and give some degree of motor strength to that concept. Therefore, if different concepts have different degrees of motor strength, this may also be the case with visual representations of concepts. The following section discusses the activation of the motor system as a result of looking at some static images.

## 5 Activation of the motor system as a result of looking at some static images

Findings of some empirical studies in the past two decades have provided evidence suggesting that the experience of looking at some static images can activate the motor system. This can be the case even with those images that do not contain any motion-related activity. As mentioned, some of these studies have found evidence suggesting that the motor system is activated when an observer looks at static letters (e.g., Longcamp et al., 2003; James and Gauthier, 2006; Wamain et al., 2012). Longcamp et al. (2011) examined the neural activities during perceiving handwritten vs printed letters. The findings of their experiment indicated that visual perception of handwritten letters results in stronger activation in the left primary motor cortex and the supplementary motor region. This stronger activity can be the result of simulating those hand movements that produce handwritten letters.

A related line of research has investigated the activation of sensory-motor areas during perception of abstract works of art. Umiltà et al. (2012) examined neural activities with electroencephalography when a group of participants were looking at paintings produced by Lucio Fontana. The mu rhythm was suppressed while observing these abstract works, providing evidence suggesting that the cortical motor system was activated during that period. Sbriscia-Fioretti et al. (2013) used ERPs to examine the involvement of sensorimotor cortical circuits while observing images of abstract works of art with marked traces of brushstrokes. Their results indicated that premotor and motor cortical areas were activated during observation.

One question raised here is “how can visual perception of a static image be the cause of activation in motor areas of the brain?” One possible answer is the association between an image and past experiences of the observer. For example, if an observer has had the experience of looking at a running cheetah, seeing the static image of a cheetah’s running posture may lead to a simulation of a running cheetah. Similarly, looking at handwritten letters can be the cause of simulating those hand movements that produce handwritten letters. In contrast, printed letters are produced by pressing buttons. The way that the printed form of letter *S* is produced does not differ from the way the printed form of letter *F* is produced. However, the hand movements that produce the handwritten form of *S* are different from the hand movements that produce the handwritten form of *F*. When a writer produces handwritten letters, s/he needs to monitor her/his hand movements to produce the right letter because a very slight deviation in hand movements can lead to producing a shape that would be very different from the intended letter. This degree of focus and monitoring hand movements is unnecessary for producing printed letters as all of them are produced by very similar movements of pressing buttons. In other words, although both handwritten letters and printed letters are produced by hand movements, the nature of hand movements involved in producing handwritten letters is different from the nature of hand movements involved in producing printed letters. That is why handwritten letters have a higher degree of motor strength than printed letters.

To take another example, looking at the image of a running track or a road with painted lines can lead to activation in the motor areas of the brain. Many people have had the experience of moving on such tracks and roads (or have seen other people running on tracks and roads). Therefore, looking at the image of a running track or a road with painted lines can lead to simulating those past experiences, because people regularly simulate and anticipate the movement/feedback they will produce. In this way, looking at such images can reactivate past motor experiences associated with them. However, this does not mean that someone who has not had the experience of running on a road would be unable to simulate her/his running on a road. Human beings have strong imaginative power and can simulate even impossible situations. Past experiences are an important part of simulation but they are not the whole of the story.

Automatic activation of motor areas as a result of looking at some objects or images of those objects has been examined by studies that have worked on affordances – the opportunities for actions or action capabilities that are offered to an agent by the environment (e.g., Gibson, 1979; Thomas et al., 2019; Borghi, 2021). For example, a road offers people the opportunity to walk on it, and a chair offers the opportunity to sit on it. Some views hold that affordances are the result of long-term visuomotor associations and are activated automatically without any relation to previous intention to act (Borghi, 2021). However, the automatic activation of affordances has been seriously challenged by studies that have found evidence suggesting that affordance activation is dependent on the task, context, and intention of the individual (Bub et al., 2018a; Xiong et al., 2019; for a review, see Chong and Proctor, 2020).

In one of these studies, Bub et al. (2008) used object priming to show that volumetric gestures (i.e., handling related to the volume of an object) and functional gestures (i.e., handling related to the conventional use of an object) can be evoked in different situations depending on the task and intention. For example, the individual may have the intention to move a calculator or to use it for calculation. In the former case, s/he might perform a unimanual action by five fingers to pick up the calculator (volumetric gesture); in the latter case, s/he might perform a bimanual action by keeping the calculator in one hand and using the pointer finger of the other hand to press the buttons (functional gesture). In another study, Bub et al. (2018b) found that lift actions were faster for objects that were targeted for a prospective use action than for objects that were not targeted for a prospective use. In other words, the intention to use an object speeded up the prior action of lifting it. Based on these findings, they suggest that this happens because the motor sequence of lifting the object and then using it is habitually conscripted to enact the proper function of an object. This means that when people look at objects, they simulate only anticipated actions or the feedbacks they will produce not all possible actions that can be done with that object. For example, when someone looks at a glass, the action of grasping the glass is simulated if this action is anticipated. This is the crucial difference between this view and the strong embodied approach, which holds that the mere experience of looking at a glass can lead to simulating the action of grasping the glass regardless of individual’s intentions, conditions, and the context.

Most of the works on affordances activation have been conducted within the framework of ideomotor theory. According to this theory, any action is represented by its perceivable effects, and there is a firm link between internal representation of an action and the action itself (Shin et al., 2010). However, as mentioned, recent evidence strongly suggests that the activation of internal representations of actions is dependent on the features of the task and the intention of the individual. In the following section, motor strength of visual representations of some scientific concepts are discussed. But, before that, it should be noted that regardless of whether affordance activation is automatic or dependent on individual’s intention/goal, visual representations can have some degree of motor strength. In other words, the main argument is that processing a visual representation can activate the motor system regardless of whether this processing is oriented or not oriented to a goal/intention.

## 6 Motor strength of visual representations

As mentioned, the process of looking at some images or visual representations may lead to the activation of motor areas in the brain. For example, looking at static handwritten letters or looking at some static abstract works of art may activate motor areas. This motor activation may be an indication that the hand movements that produce those images or visual representations are simulated by the observer. The visual representation of the mathematical function *f(x)* = *sin x* is a curve that oscillates between −1 and 1 along *y* axis and between −∞ and + ∞ along the *x* axis. The mathematical metaphor *f(x) oscillates between* −*1 and 1* describes this function in terms of the movement of an object that oscillates between the two extreme points of −1 and 1. From the perspective of the strong version of embodiment (Gallese and Lakoff, 2005), since this function is described in terms of an oscillating movement, the same cognitive resources that are employed during observing the oscillation of a moving object are also employed to process this mathematical metaphor. In other words, the motor system may be employed to simulate the hand movements that are used to depict the shape of the graph, although no gesture is involved. Therefore, processing this metaphor through its visual representation can involve the activation of motor areas in the brain. The visual representation of this mathematical function has a high degree of motor strength as people have had the experience of looking at the oscillating movement of objects.

In metaphorical descriptions of scientific concepts, sometimes fictive motion is used to ground and understand abstract and static concepts. A fictive motion metaphor attributes the feature of movement to a static concept (Talmy, 1996). The mathematical metaphor *the function f(x) approaches its maximum point as x approaches 1* is a fictive motion metaphor that describes the algebraic representation of a function and its static graphic representation as a moving object. The cognitive processing of fictive motion metaphors has been the subject of many studies from a variety of perspectives (e.g., Lakoff and Núñez, 2000; Matlock et al., 2005; Richardson and Matlock, 2007; Khatin-Zadeh and Yazdani-Fazlabadi, 2023; for a review, see Huette and Matlock, 2016). The findings of several behavioral studies suggest that processing fictive motion metaphors involves a mentally simulated motion (Boroditsky and Ramscar, 2002; Matlock, 2006; Núñez et al., 2006; Matlock et al., 2011; Rosa et al., 2020). Matlock (2010) suggests that during understanding fictive motion metaphors, people experience a fleeting sense of motion. Two neuroimaging studies have provided evidence suggesting that processing fictive motion metaphors activates a motor area in the brain that responds to perceived motion (Wallentin et al., 2005; Saygin et al., 2010). A later neuroimaging study found that the excitability of the motor system modulated by the motor component of the verb is preserved in the processing of fictive motion sentences (Cacciari et al., 2011; see also Cacciari and Pesciarelli, 2013).

If processing fictive motion metaphors creates a sense of fleeting motion and activate motor areas in the brain, visual representations of such statements can have a similar effect. This is particularly interesting in the process of transforming an abstract mathematical concept into a visual representation. Transforming algebraic representation of a function (in the form of an algebraic formula) into a visual representation in a Cartesian plane (in the form of a curve) is an example in which a mathematical entity is translated into a visual representation. The curve may have some degree of motor strength if it is like the trajectory of moving objects that a person has seen in daily experiences. The idea of motor strength of curves is supported by those studies that have found evidence suggesting that some visual representations such as handwritten letters and paintings may have some degree of motor strength (e.g., Kourtzi and Kanwisher, 2000; Senior et al., 2000; Kim and Blake, 2007; Williams and Wright, 2009; Osaka et al., 2010; Longcamp et al., 2011; Lorteije et al., 2011; Umiltà et al., 2012). These findings suggest that when someone looks at handwritten letters or a painting, s/he simulates the hand movements that have been involved in the creation of those letters or painting. This mental simulation can give the image of handwritten letters and painting some degree of motor strength.

In scientific discussions, some concepts are represented by vectors. In physics, the concepts of force, speed, and acceleration are represented by vectors. In mathematics, ordered pairs and ordered triples are represented by vectors in two dimensional and three-dimensional Cartesian coordinate systems, respectively. Vectors in various branches of mathematics represent the concepts of symmetry and one-to-one correspondence. Vector is a visual representation that has a high degree of motor strength. In people’s daily interactions, it is recurrent to show the movement of objects by vectors. The reason is that every vector has a starting point, an ending point, and a clear route. Therefore, vector is an excellent visual representation for describing the movement of objects, as it clearly shows the key components of every movement: source, goal, and route. Understanding even highly abstract scientific concepts through these motorized visual representations involves the activation of motor areas as one of the components of the cognitive system, even though there might be no noticeable body movement in the process of understanding.

## 7 Distributed nature of motor strength

Motor strength of a concept may have a distributed nature across various body parts, as the concept may have a variety of degrees of motor associations with various body parts. As mentioned, a given concept may have a strong motor association with a certain part of the body and a less strong association with some other parts of the body. In such cases, the sum of degrees of strength in various parts of the body determines degree of motor strength of that concept. Usually, the distribution of motor strength of concepts across various body parts is imbalanced. For example, the concept of *run* has a high degree of motor strength in leg, as the action of running involves the active role of legs. However, since the action of running also involves some degree of hand movements, the concept of running can have some degree of arm strength. The mouth is much less involved in the action of running. Therefore, we can expect degree of mouth strength of this concept to be very weak or almost zero. To take another example, when the concept of *quitting a habit* is metaphorically described in terms of *kick a habit*, a significant degree of motor strength is metaphorically given to the concept of *quitting*, as the verb *kick* has a high degree of motor strength in leg. In this case, the concept of *quitting* acquires a high degree of motor strength in leg but a much lower degree of motor strength in arms, head, and other parts of the body.

## 8 Conclusion

The role of the motor system in understanding a concept is dependent on the degree of motor strength of that concept. If a concept is understood in its literal sense, the employment of the motor system and gestures in processing that concept depends on its degree of motor strength. If a concept is understood in its metaphorical sense, the employment of the motor system and gestures depends on the degree of motor strength of the base concept through which that concept is understood. The degree of motor strength of a concept relies on semantic features of that concept as well as on the associations between that concept and people’s experiences related to body (or object) movements. This is also the case with metaphorical description of scientific concepts in terms of visual representations.

Introducing the notion of motor strength of concepts can offer a clearer understanding of how the motor system can contribute to the grounding of abstract concepts in the physical environment. The role of the motor system in the grounding of concrete and abstract concepts into the physical environment has been demonstrated by past research. But, what we emphasized here is that level of importance of the motor system in the grounding and the processing of a concept is largely dependent on the degree of motor strength of the concept. In fact, it is the motor strength of a concept that determines level of importance of the motor system in grounding it in the physical environment. The greater the motor strength of a concept, the greater the role of the motor system in grounding it in the physical environment and creating a sensorimotor representation of it.

Degrees of motor strength of various visual representations may range extensively. The metaphorical understanding of those concepts that are understood in terms of strongly motorized visual representations involves active employment of the motor system. The prevalence of motion-based metaphors in daily and scientific discourse indicates that motion events and the motor system are facilitating tools that effectively contribute to the process of grounding and comprehending concepts. Degree of motor strength of a concept or a visual representation is important (ordinary or scientific) because it determines to what extent body movements and the motor system can be employed to process that concept and acquire knowledge about it. Concepts or visual representations with higher degrees of motor strength can be learned more efficiently by the active employment of body movements and the motor system. This is supported by the findings of studies that have demonstrated how gesture can enhance the process of learning sciences. That is why body-based instruction with a focus on body movements can be an efficient approach for enhancing the process of learning in the education systems, particularly in the case of science education. Finding accurate methods for determining the degree of motor strength of visual representations and identifying those factors that affect degree of motor strength is a challenging question that needs to be dealt with in future research projects.

## Data availability statement

The original contributions presented in this study are included in this article/supplementary material, further inquiries can be directed to the corresponding author.

## Author contributions

OK-Z wrote the first draft of the manuscript. JH and DF commented on it and revised it. All authors contributed to the article and approved the submitted version.
